# Comparison of various techniques for the extraction of umbelliferone and herniarin in *Matricaria chamomilla* processing fractions

**DOI:** 10.1186/s13065-017-0308-y

**Published:** 2017-08-05

**Authors:** Maja Molnar, Nikolina Mendešević, Drago Šubarić, Ines Banjari, Stela Jokić

**Affiliations:** Faculty of Food Technology Osijek, Josip Juraj Strossmayer University of Osijek, Franje Kuhaca 20, 31000 Osijek, Croatia

**Keywords:** Chamomile fractions, Herniarin, Umbelliferone, Extraction, Antioxidant activity

## Abstract

Chamomile, a well-known medicinal plant, is a rich source of bioactive compounds, among which two coumarin derivatives, umbelliferone and herniarin, are often found in its extracts. Chamomile extracts have found a different uses in cosmetic industry, as well as umbelliferone itself, which is, due to its strong absorption of UV light, usually added to sunscreens, while herniarin (7-methoxycoumarin) is also known for its biological activity. Therefore, chamomile extracts with certain herniarin and umbelliferone content could be of interest for application in pharmaceutical and cosmetic products. The aim of this study was to compare the extracts of different chamomile fractions (unprocessed chamomile flowers first class, processed chamomile flowers first class, pulvis and processing waste) and to identify the best material and method of extraction to obtain herniarin and umbelliferone. Various extraction techniques such as soxhlet, hydrodistillation, maceration and supercritical CO_2_ extraction were used in this study. Umbelliferone and herniarin content was determined by high performance liquid chromatography (HPLC). The highest yield of umbelliferone (11.80 mg/100 g) and herniarin (82.79 mg/100 g) were obtained from chamomile processing waste using maceration technique with 50% aqueous ethanol solution and this extract has also proven to possess antioxidant activity (61.5% DPPH scavenging activity). This study shows a possibility of potential utilization of waste from chamomile processing applying different extraction techniques.

## Background

Cultivation of medicinal and aromatic plants, especially chamomile (*Matricaria chamomilla*), has increased in recent years and large areas of Republic Croatia are designed specifically for this type of farming. Chamomile belongs to those drugs that experienced a wide medical application, mainly due to its anti-inflammatory, antiseptic and antispasmodic activity. Application fields of chamomile products include dermatology, stomatology, otolaryngology, internal medicine, in particular gastroenterology, pulmology, pediatry and radiotherapy [1]. Chamomile extracts can also be used in different industries, which usually utilize only some parts of the plant and the rest is considered as waste.

Chamomile contains a large number of therapeutically interesting bioactive compounds, sesquiterpenes, flavonoids, coumarins and polyacetylenes being considered the most important ones [2, 3]. In existing papers that deal with the content of chamomile coumarin compounds, seven coumarins (herniarin, umbelliferone, coumarin, isoscopoletine, scopoletine, esculetin, and fraxidin) were described [4–6], while Petrulova-Poracka et al. [7] have found skimmin, daphnin, daphnetin in anthodia and leaves. Plant coumarins, in general, are usually described as phytoalexins and are considered as plant defence compounds in biotic and abiotic stress conditions [8, 9]. Content of herniarin and umbelliferone, as secondary metabolites in chamomile leaf rosettes, was proven to be higher when plant is subjected to abiotic stress [10] and Petrulova-Poracka et al. [7] found that umbelliferone in chamomille leaves is usually present in higher levels compared to anthodia (plant head). In addition, chamomile flowers also contain several coumarin compounds, herniarin and umbelliferone [7, 11–13], usually herniarin in greater amount compared to umbelliferone [14]. Redaelli et al. [14] investigated different parts of chamomile flower heads for herniarin and umbelliferone content and found that ligulate florets exhibit higher content of coumarins than other parts of the flower head.

Coumarin-related compounds exhibit antimicrobial and anti-inflammatory activity [15], while umbelliferone itself exhibits various biological properties, antioxidant activity in vitro, inhibition of HIV-1 replication and inhibition of cell proliferation of different human tumor cell lines [16, 17]. Umbelliferone is often used in sunscreens as it strongly absorbs ultraviolet light at several wavelengths [18]. Herniarin is also well known for its various biological activities [19].

Bioactive compounds are often present in the plants in low concentration and are chemically sensitive. So it is very important to investigate the effectiveness of extraction method to recover these compounds from plant material [11], especially those parts that are considered as waste from chamomile processing. The traditional methods for the extraction of plant materials include steam distillation and organic solvent extraction using percolation, maceration or Soxhlet techniques [20]. In addition, there is a growing interest in alternative extraction technologies consuming less organic solvents, due to their toxicity and regulatory restrictions. One such “green technology” is supercritical carbon dioxide (CO_2_) extraction which exhibit several advantages in the extraction of natural products from plant matrices. Extracts obtained using CO_2_ as the extraction solvent are solvent-free/without any trace of toxic extraction solvents, with better retention of aromatic compounds, and are thereby highly valued [21].

A number of studies have reported the supercritical fluid extraction (SFE) of chamomile [20, 22–30] and most of the authors investigated composition of chamomile flowers [14, 20, 26], while in this study we examined different chamomile fractions, containing different parts of chamomile, obtained during chamomile processing. These fractions include unprocessed chamomile flowers first class, processed chamomile flowers first class, pulvis and processing waste, respectively.

The various extraction techniques (soxhlet, hydrodestillation, maceration, supercritical CO_2_ extraction) were used for obtaining chamomile extracts which were further compared on the extraction yield, their antioxidant activity and umbelliferone content determined by high performance liquid chromatography (HPLC).

## Materials and methods

### Chemicals

The purity of CO_2_ used for extraction was 99.97% (*w*/*w*) (Messer, Osijek, Croatia). DPPH and ethyl acetate were purchased from Sigma-Aldrich Chemie (Steiheim, Germany). Umbelliferone and herniarin were purchased from Dr. Ehrenstorfer GmbH (Augsburg, Germany) and standard purity was 99.9% as informed by supplier. All solvents were of analytical grade and purchased from J.T. Baker (PA, USA).

### Plant material

**Fig. 1:**
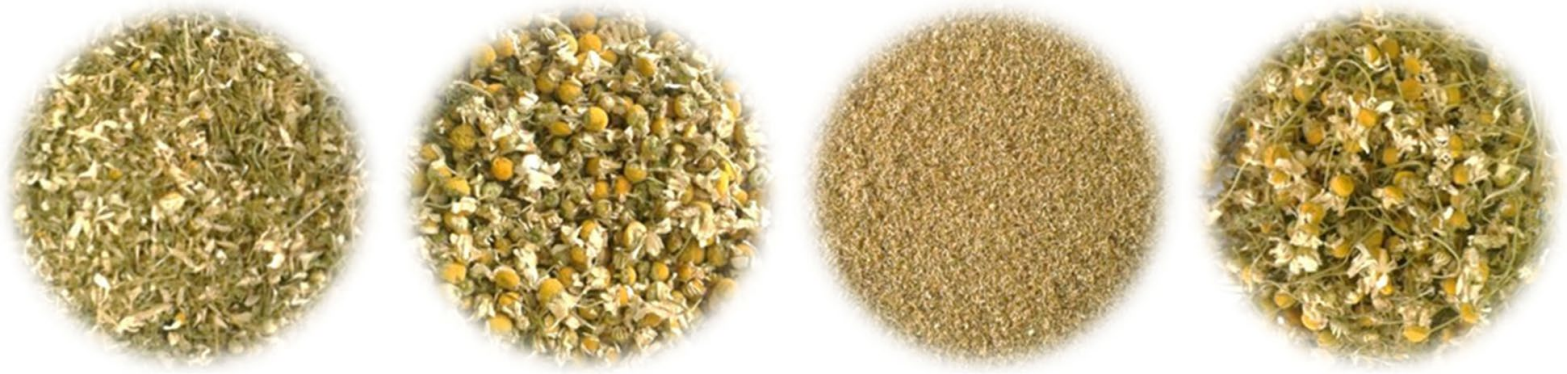
Chamomile samples used in this study (**a** unprocessed chamomile flowers first class; **b** processed chamomile flowers first class; **c** pulvis; **d** processing waste)

The following samples of chamomile (Fig. 1) were used: unprocessed chamomile flowers First class, processed chamomile flowers first class, pulvis and processing waste obtained from the company Matricia Ltd. (ŠirokoPolje, Croatia) in year 2015.

*Unprocessed chamomile flowers first class* (Fig. 1a) are related to the samples obtained after cutting fresh chamomile using machine for cutting herbs.

*Processed chamomile flowers first class* (Fig. 1b) are obtained after cutting the stems from picked chamomile flowers. High capacity sieve separates flower heads from stems and pulvis. After that, samples are dried at temperature of around 30 °C. The final product is a good-quality flowers without stems, with excellent shape and appearance.

*Processing waste* (Fig. 1c) are remaining after chamomile processing (without chamomile flower heads).

*Pulvis* (Fig. 1d) are flower parts released from the flower heads during manipulation, after the drying process.

Prior to extraction, the plant material was grounded using laboratory mill.

### Extraction procedures

#### Soxhlet extraction

A sample of 5.0 g of each plant material was extracted by 150 mL *n*-hexane using a Soxhlet apparatus until totally depleted. The whole process took 8 h. Furthermore, the solvent was evaporated under vacuum, and the obtained extracts were stored in a glass bottles at 4–6 °C. The measurements were performed in triplicate.

#### Maceration

The 20.0 g of each dried grounded material were immersed into 100 mL of 50% aqueous ethanol solution. The system was left to soak for 5 days in the dark at room temperature and it was occasionally shaken. The alcoholic extract was then filtered through filter paper to eliminate any solid impurities and concentrated in rotary vacuum evaporator at 35 °C yielding a waxy material. Finally, the extracts were kept in the dark at 4–6 °C until tested. The measurements were performed in triplicate.

#### Hydrodistillation

The 100 g of each samples were used for hydrodistillation (4 h) in Clevenger type apparatus. The essential oil was dried over anhydrous MgSO_4_ and kept at 4–6 °C until further analysis. The measurements were performed in triplicate.

#### Supercritical CO_2_ extraction

The experiment was performed in SFE system explained in detail previously [31]. Each chamomile sample (100 g), respectively, was placed into the extractor vessel and the extracts were collected in a separator in previously weighed glass tubes at 1.5 MPa and 25 °C. The amount of extract obtained at regular intervals of time was established by weight using a balance with precision of ±0.0001 g. Extraction yield was expressed as % (g of extract/100 g of dried material). The extraction was performed at extraction conditions of 30 MPa and 40 °C. Dynamic extraction mode for SFE was used where supercritical CO_2_ continuously passed through the sample matrix (chamomile). The mass of dried material in extractor, the extraction time and CO_2_ mass flow rate were kept constant during experiments. The CO_2_ flow rate (2 kg/h) was measured by a Matheson FM-1050 (E800) flow meter. Each extraction run lasted for 90 min, since longer extraction times did not significantly increase the extraction yield (based on our preliminary experiments). The obtained extracts were kept at 4–6 °C until HPLC analyses. The measurements were performed in triplicate.

### Determination of umbelliferone and herniarin concentration by HPLC

**Fig. 2:**
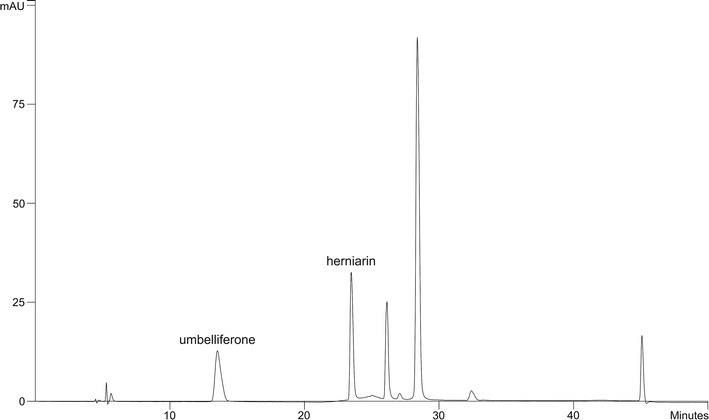
HPLC chromatogram of chamomile extract

RP-HPLC method with UV detection was used for umbelliferone and herniarin determination in obtained extracts according to the application for used column. The example of HPLC chromatogram of the extract from processing waste obtained by Soxhlet technique is given at Fig. 2. HPLC analyses were performed on a Varian ProStar system (Varian Analytical Instruments, CA, USA) consisted of Varian ProStar 230 Solvent Delivery Module, ProStar 500 Column Valve Module and ProStar 330 Photodiode Array detector. System was coupled to a computer with the ProStar 5.5 Star Chromatography Workstation and PolyView 2000 V 6.0.

Chromatographic separation was obtained on a COSMOSIL 5C18-MA-II (NacalaiTesque, Inc., Kyoto, Japan) column, 150 mm long with internal diameter of 4.6 mm.

Separation of analysed compounds was performed with gradient elution where distilled water was used as phase A and methanol as phase B. The following gradient was used: 0–15 min, 60% A and 40% B phase; 15–20 min, increasing the share of phase B to 80% and decreasing phase A to 20%; 20–40 min, holding 20% A and 80% B phase; 40–41 min decreasing of B phase to 40% and increasing A phase to 60%, 41–50 min, holding 60% A and 40% B phase. The flow rate was 1.0 mL/min, injection volume was 20 µL, UV detection wavelength 330 nm and chromatography was performed at room temperature. Standard stock solutions were prepared in a solvent and calibration was obtained at six concentrations (concentration range 1.0, 2.0, 5.0, 10.0, 20.0, 50.0 mg/L). Linearity of the calibration curve was confirmed by R^2^ = 0.9996 for umbelliferone. Umbelliferone limit of detection (LOD) was 0.16 mg/L, limit of quantification (LOQ) was 0.52 mg/L and compound retention time was 13.37 min. Linearity of the herniarin calibration curve was confirmed by R^2^ = 0.9999. Herniarin limit of detection (LOD) was 0.129 mg/L, limit of quantification (LOQ) 0.4299 mg/L and compound retention time was 24.72 min. Extracts were diluted in methanol HPLC grade, filtered through 0.45 μm PTFE filters and subjected to HPLC analyses.

Concentration of umbelliferone and herniarin in plant extracts (μg/mL) determined by HPLC analysis was used for calculation of their yield expressed as mg of compound/100 g of chamomile sample.

### Determination of antioxidant activity

Antioxidant activity of chamomile extracts was determined using DPPH method described earlier [32]. Plant extracts were dissolved in methanol (125 μg/mL) and mixed with 0.3 mM DPPH radical solution. The measurements were performed in triplicate.

The absorbance was measured at 517 nm and DPPH scavenging activity was determined using Eq. (1):

1 $$\% \;DPPH\;activity = \frac{{\left( {A_{DPPH} + A_{b} } \right) - A_{s} }}{{A_{DPPH} }}*100$$

### Statistical analysis

One-way analysis of variance (ANOVA) and multiple comparisons (Duncan’s post hoc test) were used to evaluate the significant difference of the data at *p* < 0.05. Data were expressed as means of replication ± standard deviation.

## Results and discussion

The chamomile extracts in this study were obtained from different chamomile fractions using four extraction techniques and the results related to obtained extraction yield and antioxidant activity of obtained extracts are given in Table 1, while results for herniarin and umbelliferone content in obtained extracts are given in Table 2. The results show that there were significant differences (*p* < 0.05) between analysed chamomile fractions on all analysed variables. The ANOVA analysis of extraction yields and antioxidant activity of chamomile extracts (Table 1) showed the existence of four groups (different letter identifiers) which differed significantly from one to another (*p* < 0.05; Duncan’s post hoc test) depending on the used chamomile fraction in the case of SFE, while soxhlet and maceration techniques showed the existence of three groups which differed significantly from one to another (*p* < 0.05; Duncan’s post hoc test). Hydrodistillation show no statistically significant differences in antioxidant activity of essential oils obtained from four different fractions (one group of letter).

**Table 1:** Extraction yields and antioxidant activity of chamomile extracts

Analysed variable/sample	Extraction method			
	SFE	Soxhlet	Maceration (with 50% ethanol)	Hydrodistillation
Extraction yield (g/100 g)				
Unprocessed chamomile flowers first class	1.57 ± 0.11^a^	4.60 ± 0.24^a^	20.85 ± 0.44^a^	0.41 ± 0.06^a^
Processed chamomile flowers first class	3.64 ± 0.16^b^	4.98 ± 0.31^a^	22.30 ± 0.77^b^	0.62 ± 0.09^b^
Processing waste	0.23 ± 0.07^c^	3.47 ± 0.11^b^	20.60 ± 0.51^a^	0.24 ± 0.08^c^
Pulvis	0.97 ± 0.08^d^	1.45 ± 0.13^c^	6.70 ± 0.34^c^	0.28 ± 0.06^c^
% DPPH scavenging				
Unprocessed chamomile flowers first class	5.1 ± 0.13^a^	2.0 ± 0.14^a^	56.0 ± 0.82^a^	3.9 ± 0.10^a^
Processed chamomile flowers first class	3.4 ± 0.21^b^	1.3 ± 0.07^b^	55.0 ± 0.74^a^	3.8 ± 0.12^a^
Processing waste	4.5 ± 0.33^c^	2.5 ± 0.08^a^	61.5 ± 0.23^b^	2.9 ± 0.14^a^
Pulvis	7.2 ± 0.18^d^	0.0 ± 0.00^c^	45.4 ± 0.86^c^	3.2 ± 0.18^a^

Data are expressed as mean value of replication (n)

The same letter in the same column of analysed variable indicates no significant differences (Duncan’s test, p < 0.05)

**Table 2:** Umbelliferone and herniarin content in chamomile extracts

Analysed variable/sample	SFE	Recovery (%)	Extraction method				Hydrodistillation
			Soxhlet	Recovery (%)	Maceration (with 50% ethanol)	Recovery (%)	
mg umbelliferone/100 g							
Unprocessed chamomile flowers first class	0.00^a^	98.70	0.50 ± 0.02^a^	98.64	5.59 ± 0.05^a^	98.58	nd^a^
Processed chamomile flowers first class	0.33 ± 0.00^b^	98.32	0.00^b^	100.82	4.78 ± 0.15^b^	97.45	nd^a^
Processing waste	0.02 ± 0.00^a^	97.91	0.85 ± 0.03^a^	96.36	11.80 ± 0.17^c^	98.33	nd^a^
Pulvis	0.32 ± 0.02^b^	102.38	0.13 ± 0.02^c^	98.82	5.26 ± 0.14^a^	103.42	nd^a^
mg herniarin/100 g							
Unprocessed chamomile flowers first class	13.08 ± 1.78^a^	103.9	37.66 ± 5.46^a^	98.1	47.45 ± 5.11^a^	102.8	<LOD^a^
Processed chamomile flowers first class	37.05 ± 6.29^b^	100.2	20.22 ± 2.28^b^	93.5	45.54 ± 4.16^a^	104.0	<LOD^a^
Processing waste	2.71 ± 0.12^c^	90.8	41.18 ± 2.59^a^	103.6	82.79 ± 3.26^b^	97.6	<LOD^a^
Pulvis	15.57 ± 2.87^b^	90.6	5.63 ± 0.75^c^	95.8	20.81 ± 0.00^c^	103.1	<LOD^a^

Data are expressed as mean value of replication (n) ±SD

The same letter in the same column of analysed variable indicates no significant differences (Duncan’s test, p < 0.05)

nd, not detected; <LOD, below limit of detection

### Extraction of *M. chamomilla* processing fractions

The greatest extraction yield was obtained using maceration technique compared to other extraction methods which reduces the extraction time and provides extracts with higher antioxidant activity (Table 1). In maceration process, the ethanol was chosen as the solvent based on its environmental-friendly characteristics, low cost and its ability to enhance the extraction of target compounds from vegetable materials. Ethanol in the concentration 20–100% (*v*/*v*) is the most common organic solvent used in extraction of flavonoids, phenolics, anthocyanins, lycopene, and others, from plant materials [33]. These compounds are generally more soluble in water–ethanol solutions than in pure alcohol. The highest extraction yield in maceration process was obtained from processed chamomile flowers first class, while unprocessed chamomile flowers first class and processing waste show no significant differences (*p* < 0.05) between obtained extraction yield.

There were statistically significant differences (*p* < 0.05) between extraction yields obtained by supercritical CO_2_ from all four chamomile fractions. The highest extraction yield was obtained from processed chamomile flowers first class (3.64/100 g). Extraction yields obtained with supercritical CO_2_ were more comparable to yield obtained with *n*-hexane in Soxhlet apparatus, while maceration using 50% ethanol solution provided much higher yields. This can be explained by similar dissolving capacity of supercritical CO_2_ and *n*-hexane because both are non-polar solvents, dissolving non polar compounds only, while ethanol as a polar solvent dissolved the whole soluble polar compounds. According to that, the SFE extraction is more selective extraction technique compared to maceration. The similar conclusion is obtained by Felfoldi-Gava et al. [34] where authors published approximately 20 times higher yield of alcoholic ethanol extracts then the SFE or *n*-hexane extracts. Roby et al. [35] also compared different solvents in extraction of chamomile flowers and found that the extracting ability is as follows: methanol > ethanol > diethyl ether > hexane.

The highest essential oil content obtained by hydrodistillation in this study was 0.6% from processed chamomile flowers first class. Other chamomile fraction had lower essential oil content. The chamomile oil content is usually very low and varies from 0.3 to 1.5% [3], while Roby et al. [35] obtained 0.73%. The obtained essential oil was characterized by blue color, while SFE extracts and extracts obtained by ethanol water solution had dark yellow colour which is in accordance with previous studies [25]. Dark yellow color indicates that no thermal degradation of the naturally occurring matricine to chamazulene has occurred. Matricine is converted upon steam distillation or exposure to heat into chamazulene, a sesquiterpene responsible for the blue colour of the distillate [2, 36].

Kotnik et al. [20] investigated the supercritical CO_2_ extraction of chamomile flower heads, and the results were compared with those obtained with Soxhlet extraction, steam distillation and maceration. Extraction yields obtained conventionally by maceration with ethanol and Soxhlet extraction were higher up to 10% then the yield obtained by SFE (3.81%), while the yield obtained with distillation process was very low and similar with our study, 0.60%. Also, chamazulene was detected only in the extract obtained by steam distillation; in other extracts was not present. Scalia et al. [26] also compared SFE with conventional extraction techniques for the isolation of the active compounds present in chamomile flower heads. The yield of essential oil obtained with supercritical CO_2_ was 4.4 times higher than that produced by steam distillation, similar like in our study.

Using supercritical CO_2_ extraction, degradation of thermolabile compounds (e.g. matricine) is minimized and the yield of volatile analytes is increased. Therefore, the possibility of producing plant extracts without any contact with conventional organic solvents and thus directly usable, makes the SFE technique an attractive alternative to the other currently used methods.

### Herniarin and umbelliferone content

As *M. chamomilla* is a well-known herniarin and umbelliferone containing plant [7], many researchers have dealt with their isolation from this plant. Umbelliferone can be extracted with water [36], ethanol or aqueous ethanol [37], methanol [38], while solvents like ether or dichloromethane are not so efficient [39]. Bajerova et al. [40] compared different techniques in extraction of umbelliferone from different plants, proving that Soxhlet extraction with methanol was the most efficient one, while SFE extraction was not efficient probably due to CO_2_ being non polar solvent. This is in accordance with our findings in Table 1, where polar solvents are proven to be more efficient than non-polar ones, like *n*-hexane (Soxhlet) and CO_2_ (SFE).

The data given in Table 2 for umbelliferone content indicates that the highest umbelliferone content (11.80 mg/100 g) were obtained from chamomile processing waste using maceration technique and aqueous ethanol solution as a solvent. Also, the highest herniarin content (82.79 mg/100 g) was found to be in chamomile processing waste extract obtained by the same maceration technique. A high umbelliferone and herniarin content in the extracts obtained by maceration technique can be explained by the fact that these samples which remain after chamomile processing are mainly steam and leaves, which are also rich in these compounds, often more than flowers [7]. In the essential oils of all four chamomile fractions obtained by hydrodistillation, herniarin and umbelliferone were not detected.

The ANOVA analysis of umbelliferone and herniarin content of chamomile extracts (Table 2) showed the existence of mainly three groups which differed significantly from one to another (*p* < 0.05; Duncan’s post hoc test) depending on the used chamomile fraction; only in the case of hydrodistillation there were no statistically significant differences because umbelliferone content was not detected and herniarin content was below limit of detection (<LOD) in all analysed chamomile fractions.

### Antioxidant activity of obtained extracts

Furthermore, these chamomile extracts (Table 1) have also proven to possess antioxidant activity (45.4–61.5% DPPH scavenging activity). This was expected, since polar solvents are more effective in extraction of polar compounds, like polyphenols, which greatly contribute to antioxidant activity. Bajerova et al. [40] also found that extracts of chamomile obtained with polar solvents possess better antioxidant activity than SFE extracts. Also, Formisano et al. [41] compared antioxidant activity of methanolic chamomile extracts and essential oil and found that methanolic extracts showed much better activity than essential oils, presuming that methanolic extracts are richer in phenols, thus contributing to antioxidant activity. This was also observed in our investigation, where SFE extracts did not show any significant antioxidant activity and neither did the hexane extracts, which is expected, since CO_2_ and hexane possess a similar dissolving capacity. The antioxidant activity of essential oils obtained by hydrodistillation was also low and not comparable to ethanol extracts.

## Conclusions

Processing waste which remains after chamomile processing in significant amounts can be considered as a rich source of coumarin derivatives—herniarin and umbelliferone. Umbelliferone is often used in cosmetic industry due to its strong absorption of UV light and for its extraction from plant material different extraction techniques can be employed. Hereby, in this research we compared SFE, hexane and ethanol extraction (maceration) and hydrodistillation and proved that aqueous ethanol is the most effective in this regard. These extracts not only had the highest umbelliferone and herniarin content, but also showed a significant antioxidant activity. For potential utilization in cosmetic industry it would be interesting to obtain extracts with high umbelliferone and herniarin content and antioxidant activity as additives to different cosmetic products.
